# 

**Published:** 1915-12

**Authors:** 


### CONTENTS, DECEMBER, 1915—VOLUME XXXI, NO. 6.

### ORIGINAL ARTICLES.

Hypo and Hyperthyroidism. By J. S. Hixson, M. D., San Angelo, Texas ........ 223

Dislocation of Elbow Joint and Flexed Ankylosis of the Knee Joint. By Joe Gilbert, M. D., F. A. C. S., Austin, Texas.... 226

Symptoms of Chronic Appendicitis. By Wade H. Walker, M. D., Wichita Falls, Texas........ 229

Recurrent Bronchitis. Dr. L. P. Tenny, Troup, Texas......... 231

The Mystery of Infection. By Dr. G. Henri Bogart, Shelbyville, Ind. ........ 234

### DEPARTMENT OF EUGENICS.

Infant Mortality and Natural Selection........ 236

The Effects of Selection........ 236

Biology to the Fore Again........ 237

Short Cut to Eugenics........ 238

Sane at Last........ 240

### EDITORIAL DEPARTMENT.

Medical Editors' Association........ 243

Public Health Service Discovers Cause and Cure of Pellagra...... 245

The American Society for the Study of Alcohol and Other Narcotics. 247

Seize Substitute Specifics........ 248

Report of Clinic on Pellagra Held in Dallas, Texas, in Connection With Meeting of Southern Medical Association, November 8-11, 1915. By John R. Lehmann, A. B., M. D., Dallas..... 249

Scintillations from the Pellagra Symposium........ 253

Dallas Clinics ........ 255

Items of General Interest........ 258

Personal Mention ........ 260

Helpful Hints for the Busy Doctor........ 262

